# Futuristic Clothes: Electronic Textiles and Wearable Technologies

**DOI:** 10.1002/gch2.201900092

**Published:** 2020-03-18

**Authors:** Ezgi Ismar, Senem Kurşun Bahadir, Fatma Kalaoglu, Vladan Koncar

**Affiliations:** ^1^ Nano Science & Nano Engineering Istanbul Technical University Istanbul 34467 Turkey; ^2^ Department of Mechanical Engineering Istanbul Technical University Istanbul 34437 Turkey; ^3^ Department of Textile Engineering Istanbul Technical University Istanbul 34437 Turkey; ^4^ GEMTEX University of Lille Cité Scientifique Villeneuve d'Ascq F‐59650 France; ^5^ École Nationale Supérieure des Arts et Industries Textiles/Génie et Matériaux Textiles laboratory (ENSAIT/GEMTEX) 2 Allée Louis et Victor Champier Roubaix F‐59100 France

**Keywords:** conductive textiles, e‐textiles, flexible electronics, smart textiles, wearable electronics

## Abstract

This review summarizes the recent developments and importance of wearable electronic textiles in the past decade. Wearable electronic textiles are an emerging interdisciplinary research area that requires new design approaches. This challenging interdisciplinary research field brings together specialists in electronics, information technology, microsystems, and textiles to make an innovation in the development of wearable electronic products. Wearable electronic textiles play a key role among various technologies (clothing, communication, information, healthcare monitoring, military, sensors, magnetic shielding, etc.). In this review, applications of wearable electronic textiles are described, including an investigation of their fabrication techniques. This review highlights the basic processes, possible applications, and main materials to build wearable E‐textiles and combines the fundamentals of E‐textiles for the readers who have different backgrounds. Moreover, reliability, reusability, and efficiency of wearable electronic textiles are discussed together with the opportunities and drawbacks of the wearable E‐textiles that are addressed in this review article.

## Graphical abstract

Conventional textile structures are converted to functional surfaces and materials. From those materials, smart and wearable electronic apparels are created, which become a source of artificial intelligence and big data. The current status of wearable electronic textiles and their fabrication techniques are presented together with their impacts, drawbacks, challenges, and possible application areas.

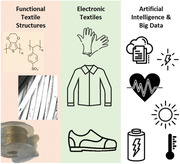

## 1 Introduction

Wearable E‐textiles is an interdisciplinary field where the basic related concepts are highlighted, aggregated, and presented for the readers in this review article. Also, concerns regarding possible future obstacles to E‐textiles are discussed in the later sections. In the literature, review articles belonging to this subject are readily available. However, the novelty and necessity of this review article can be explained as follows: it brings the latest studies up to date, makes it easy for the readers from different disciplines, and presents it in a comprehensible way.

Traditional textiles simply function as a covering material. Based on the rapidly changing global demands and in the light of technological improvements, the development of responsive functionality on textiles has led to the emergence of smart textiles accommodating the revolution we are seeing in the field of wearable electronics.

Smart textiles which are also named intelligent textiles differ from the functional textiles and should not be confused. While functional textiles provide functionally in terms of the addition of material, finishing, etc. the smart textiles are able to react after the interpretation of data generated by conditions/stimuli due to their nature.^[^ ^1^, ^2^ ^]^ Smart textiles differ from conventional textiles in terms of sensing and responding to their environment.^[^ ^3^ ^]^ They can be described as materials which can sense and react to environmental conditions or stimuli according to thermal, mechanical, electrical, magnetic, or other bases.^[^ ^4^ ^]^ They can be divided into two major categories namely i) active, and ii) passive smart textiles. Passive smart textiles can change their situation according to environmental stimuli while active smart textiles are equipped with sensors and actuators that can detect several signals from the environment and then give a meaningful response.^[^ ^3^, ^5^ ^]^ Lots of exciting applications and materials in various fields have demonstrated the transformation of conventional textiles.^[^ ^4^ ^]^

With the developing technologies and worldwide digitalization, clothes have started to become a novel type of high‐tech product via the combination of electronics. Electronic textiles are an emerging technology that necessitates new design approaches cutting across traditional industrial boundaries. This challenging interdisciplinary field of research brings together specialists in information technology, microsystems, and textiles in order to make a breakthrough in the development of wearable local monitoring, computation systems, and wireless communication applications.^[^ ^6^, ^7^ ^]^

The birth of electronic textiles incorporates interdisciplinary studies such as textiles, nano/micro technologies, computing systems, and communications and information technologies.^[^ ^8^ ^]^

At the beginning of electronic textile products, there was a huge challenge on how to reuse them without losing their properties. One of the most important challenges about E‐textiles is to make them reusable and efficient enough and to overcome the washing process making them durable through their life cycle and reusable.^[^ ^9^, ^10^, ^11^, ^12^ ^]^ Another difficulty of electronic textiles is how to combine the appropriate materials together to provide the desired features and create hybrid materials from the view of the perspectives of textile and electronic aspects.^[^ ^13^ ^]^

Electronic textiles have been emerging as solutions to serve humanity in terms of comfort, making life easier, and addressing safety issues. The main study areas of E‐textiles are the electronics industry, communication systems, military applications, the entertainment sector, and the health industry. Healthcare systems, in providing wearable electronic products offer ease of use while ensuring help, diagnosis, even rehabilitation, and therapy.^[^ ^14^, ^15^ ^]^

Textile products such as fibers, yarns, fabrics—and even their manufacturing systems—have their flexibilities which offer specialization in their desired features for a variety of product applications. Therefore, the interdisciplinary research work textile products can be transferred to be used as an information‐processing infrastructure thanks to their ability of feel, sense, and take action according to user stimuli and/or environmental factors.^[^ ^16^ ^]^

The most remarkable form of wearable electronics is wearable antennas due to increased demand in wireless technology in the communications arena.^[^ ^17^ ^]^ Wearable sensors improve the chances for survival by recognizing emergencies at home due to their connections with information and monitoring systems.^[^ ^18^ ^]^

The attachment of electronic components into garments, satisfying electronic textile‐based architecture builds new features for conventional textiles, however, preserving expected textile physical properties is a critical issue for the properties of clothing.^[^ ^19^ ^]^

In the light of the improvements to electronically conductive textiles, fibers, yarns, and fabrics functionally become sensors or electromagnetic interfaces while providing corrosion protection, monitoring and/or data transfer from cloth, electrostatic discharge, etc. Growing innovation and development in E‐textiles with novel electrical properties in conventional textiles represents an enormous field for application.^[^ ^20^ ^]^

The miniaturization in the electronics industry has led electronic components getting smaller and smaller and thus the research in embedding those components into textiles has attracted considerable interest from researchers. Recently, researchers have been seeking the ways to integrate electronic compounds into textiles in cost‐effective, reliable, and efficient ways.^[^ ^21^ ^]^

Some developed E‐textiles' prototypes are seen to be adequate for possible applications due to their unique properties such as their lightweight, comfort and flexibility features while still providing functioning. However, most of the applications are still in prototype form and the mass fabrication of E‐textiles is still a challenge.^[^ ^22^ ^]^

For the creation of wearable electronics, fiber‐based electronic compositions are the most favorable ones due to their lightweight, comfort and flexibility features.^[^ ^23^ ^]^ Flexibility is one of the critical parameters from the textile perspective which makes the structure wearable. If the material is rigid, then it is not an applicable solution for clothing in terms of comfort. Textile products can be a good option to adopt electronics when the electronic systems are flexible enough to be embedded into textiles, due to their widespread use in daily life.^[^ ^1^ ^]^

Even if the smart textiles field is huge and there are more and more scientific activities related to them and, consequently, more and more scientific articles and prototypes, it is important in our opinion to continue to survey the existing situation. It may also be observed that despite the large number of academic and industrial laboratories having more or less finalized prototypes, there are very few available products on the market. This is due to a lack of standards and norms including test procedures for E‐textiles currently. This article aims at the establishment of an assessment of the existing situation in terms of prototypes and achievements of various research groups without taking into account the market ready E‐textile products. It is supposed to help associations and standardization organizations such as IPC (www.ipc.org), AATCC (www.aatcc.org), CEN (www.cen.eu), IEC (www.iec.ch), among others, to better understand the needs and problems in the area of Smart and E‐textiles. By definition, E‐textiles contain textile structures and electronic devices for sensing, actuation, communication, decision making, etc., and they are quite different. Textiles are lightweight, flexible, washable, etc. and electronic devices are fragile, delicate, and they also have to be powered.

Even if the smart textiles field is huge and there are more and more scientific activities related to them and, consequently, more and more scientific articles and prototypes, it is important in our opinion to continue to survey the existing situation. It may also be observed that despite the large number of academic and industrial laboratories having more or less finalized prototypes, there are very few available products on the market. This is due to a lack of standards and norms including test procedures for E‐textiles currently. This article aims at the establishment of an assessment of the existing situation in terms of prototypes and achievements of various research groups without taking into account the market ready E‐textile products. It is supposed to help associations and standardization organizations such as IPC (www.ipc.org), AATCC (www.aatcc.org), CEN (www.cen.eu), IEC (www.iec.ch), among others, to better understand the needs and problems in the area of Smart and E‐textiles. By definition, E‐textiles contain textile structures and electronic devices for sensing, actuation, communication, decision making, etc., and they are quite different. Textiles are lightweight, flexible, washable, etc. and electronic devices are fragile, delicate, and they also have to be powered.

## 2 Materials and Process

E‐textile sources can be abundant and have a wide range of material types: from fibers to fabrics, coatings to stitching. However, when we take a look at the key elements, generally materials are synthetic, polymer‐based products. The basic building blocks of these materials are polymers which lead to fibers and yarns which can then be used in stitching or embroidery and then can take shape as the surface of woven, knitted, nonwoven forms. When we return to the building blocks of polymers it is also possible to choose a different approach that uses coating and printing on the textile surface with improved polymer properties. In this section, general information about the E‐textile materials is presented.

### 2.1 Conductive Polymers for Smart Textiles

**Figure 1:**
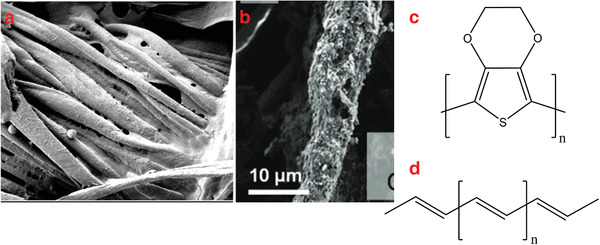
SEM image of cotton fibers with PEDOT:PSS coating. Reproduced with permission.^[^ ^28^ ^]^ Copyright 2018, MDPI. b) SEM image of PANI and carbon‐coated yarns. Reproduced with permission.^[^ ^29^ ^]^ Copyright 2016, Springer Nature. Chemical structure of c) PEDOT and d) polyacetylene.

Inherently conductive polymers can be used to create conductive fibers via melt spinning and wet spinning techniques. Moreover, through the coating of fibers with electrically conductive materials and functionalization of fibers with conductive polymers are considered as a promising approach.^[^ ^24^ ^]^ Obtaining a conductivity feature from plastics can be achieved with inherently conductive polymers due to their long‐conjugated chains which contain double pi bonds. Besides, heteroatoms can also be obtained to mix with the electrically nonconductive polymers and carbon derivatives, metal particles or electrically active polymers in order to create the conductivity feature.^[^ ^25^ ^]^ Polymers are insulator or semiconductor in their neutral or undoped form. Inherently conductive polymers have a conductivity value in the range of semiconductors to the beginning of the metallic conductors and they have a value ranging from 10^−6^ to 10^2^ S cm^−1^.^[^ ^26^ ^]^ The polymers gain electrical conductivity with oxidation or reduction reactions, that create delocalization at the backbone of the polymer chain. Conducting polymers contain a p‐electron backbone responsible for their unusual electronic properties such as electrical conductivity, low energy optical transitions, low ionization potential, and high electron affinity. This extended p‐conjugated system of the conducting polymers has single and double bonds alternating along the polymer chain. The higher electrical conductivity values obtained in such organic polymers which are named as “synthetic metals.”^[^ ^27^ ^]^ which explains that, conductive polymers have combined properties of metals and plastics. The conductive polymers are belonging to polyenes or polyaromatics. Examples of well‐known conductive polymers are; polyacetylene, polyaniline, polypyrrole, poly(3,4‐ethylene dioxythiophene), poly(p‐phenylene), polyphenylene. The chemical structure of PEDOT and polyacetylene is given in **Figure** **1**.

In situ polymerization of aniline on the textile substrates gives a chance to create electrically conductive textiles due to the efficient polymerization of aniline. Thus, textile‐based materials such as; cotton or polyacrylonitrile are easily covered with polyaniline.^[^ ^30^, ^31^, ^32^ ^]^ Moreover, in situ polymerization of pyrrole on the polyester base fabric is also available to create inherently conductive textile surfaces from inherently conductive polymers.^[^ ^33^ ^]^ Due to the ease of deposition of the polypyrrole on the wide variety of textiles, conductive textile surfaces from cellulose‐based, synthetics to wool are also obtained.^[^ ^34^, ^35^ ^]^ Conductive polymer coated textiles or films can be used in a variety of applications; from military to aviation.^[^ ^36^, ^37^ ^]^ With the light of the developed technologies, it is possible to achieve conductive polymer coated yarns and fibers.^[^ ^38^ ^]^

Generally, inherently conductive polymers have poor mechanical properties, they are insoluble and presenting nonmelting features. Thus, they offer low processability. For this reason, a combination of inherently conductive polymers with daily used polymers is required to overcome expected mechanical and physical properties. Due to this fact, related textile combinations of those conductive polymers are preferred.^[^ ^25^, ^39^ ^]^ Polymers may be a suitable choice for wearable technologies thanks to their flexibility, inherent mechanical stiffness, adaptable structure with stretchable electronic devices.^[^ ^40^, ^41^ ^]^

### 2.2 Conductive Fibers/Yarn‐Like Structures

**Figure 2:**
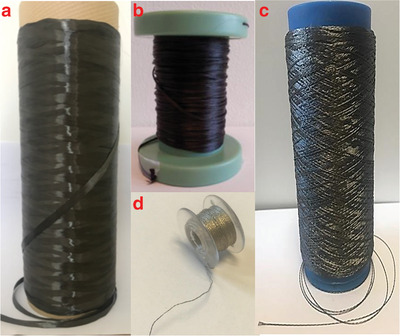
Conductive yarns: a) Carbon fiber, b) carbon‐nanotube‐containing yarn. Reproduced with permission.^[^ ^51^ ^]^ Copyright 2014, Elsevier. c) Stainless steel yarn, d) silver‐coated polyamide yarn.

Conductive yarns are available in the form of wire, staple fiber or multifilaments. They can be obtained from the electrically conductive materials such as carbon, copper, aluminum, titanium, and stainless steel or they can be obtained via coating and filling of nonconductive fibers with electrically conductive materials.^[^ ^42^ ^]^ Two practical methods are used to produce polymer‐based conductive yarns; coating and spinning techniques. During the spinning process (melt or wet), the solution contains a conductive polymer is used to obtain conductive fibers.^[^ ^43^, ^44^ ^]^ Likewise, electrically conductive materials or metal powders are used as a coating substance to cover the yarns in order to provide the conductivity feature.^[^ ^45^, ^46^ ^]^ Also, the powder form of inherently conductive polymers is mixed with polymers to obtain fibers via melt or solution spinning process.^[^ ^47^, ^48^ ^]^ **Figure** **2** shows examples of different conductive yarns.

Metal‐based yarns have a superior electrical conductivity over inherently conductive polymeric yarns. However, the sensation of metallic fibers on the skin is not very comfortable. For this reason, metallic fibers are preferred use as a conductive filler with the conventional yarns and fabrics.^[^ ^49^, ^50^ ^]^ Moreover, the stitching of metallic yarns is quite difficult compared to electrically conductive polymeric yarns.

Organic thin film transistors are compatible with E‐textiles in terms of flexibility, however, their integration with the textile structure has some challenges.^[^ ^52^ ^]^ Polyamide fibers can be covered with a thin layer of silver and it presents high electrical conductivity, while preserving the flexibility of the polyamide.^[^ ^53^ ^]^ Apart from silver‐coated polyamide yarns, insulated copper yarns have been widely used to create electrical circuits in textile structures.^[^ ^54^, ^55^, ^56^ ^]^

Thanks to the developing technology, exclusive properties of carbon nanotubes (CNTs), carbon fibers, and graphene fibers (yarn‐based structures) ensure a source for the electronic textile subtracts. CNTs and graphene have high conductivity and lightweight features which also make them suitable for electrochemical applications.^[^ ^57^ ^]^ For another well‐designed option to fabricate conductive yarns is to coat fibers with conductive polymers at their crossing points.^[^ ^58^ ^]^

Carbon nanotubes are used with the yarns to improve the yarns' strength, thermal and electrical conductivity feature.^[^ ^59^ ^]^ In an effort to manufacture conductive yarn, polyester fibers are also tried to be covered with MnO_2_ flowers and CNTs. The obtained conductivity level is promising for wearable energy storage applications.^[^ ^60^ ^]^ Also, multiwalled carbon nanotubes were coated on the textile substrates and they were successfully used as a motion sensing element.^[^ ^61^ ^]^

During the past decades, graphene gained more and more attention due to its unique properties such as thermal and electrical conductivity, flexibility, good chemical and thermal stability, and high surface area.^[^ ^62^, ^63^, ^64^ ^]^ Also, 2D graphene has been described as a component for the creation of high performance flexible conducting textiles in the form of fiber, yarn, and fabric. It is stated that those forms can be adaptable to wearable electronics. Confined graphene fibers were produced out of graphene oxide suspensions and it is reported that those fibers presented stable resistance.^[^ ^65^, ^66^ ^]^ Likewise, electrospun nylon‐6 yarns were successfully coated with reduced graphene oxide and it is stated that those yarns and nonwoven form of electrospun webs presented the acceptable conductivity feature.^[^ ^65^ ^]^

Flexible graphene fibers, which are the new class of fibers can present flexibility in high strength range electrical and thermal conductivities thus, they are ideal to be used as a flexible electrode in supercapacitor applications and can be adapted to wearable electronics in their woven forms.^[^ ^67^ ^]^ Although, direct assemble of 2D graphene layers into the fibers has some challenges, they present superiorities like low cost, lightweight and ease of functionalization compared to traditional carbon fibers.^[^ ^66^, ^68^ ^]^ Also, graphene‐based wearable E‐textiles are superior to the traditional metal‐based ones in terms of their lightweight nature. However, there are also some drawbacks to the combination of textile materials with graphene oxide, such as the complexity of their processing and difficulties in their scale‐up.^[^ ^69^ ^]^

### 2.3 Conductive Polymer Composites/Fabric Structures

#### 2.3.1 Woven and Knitted Structures

**Figure 3:**
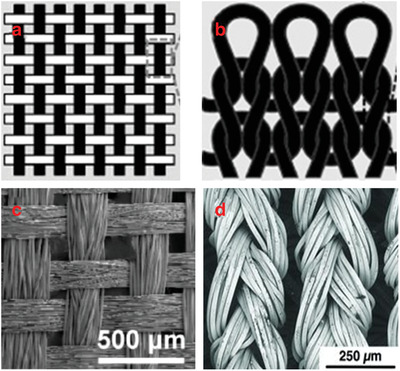
Schematic representations of a) woven and b) knitted fabric constructions. Reproduced with permission.^[^ ^83^ ^]^ Copyright 2017, Elsevier. c) The morphology of the nickel‐coated woven fabric. Reproduced with permission.^[^ ^84^ ^]^ Copyright 2013, American Chemical Society. d) Knitted fabric containing silver‐coated polyamide yarns. Reproduced with permission.^[^ ^85^ ^]^ Copyright 2012, John Wiley and Sons.

Textile substrates can be fabricated with different fabrication techniques. Fabric refers to any kind of textile material made through weaving, knitting, or nonwoven techniques. **Figure** **3** shows the basic structure of woven and knitted fabrics. All those methods have been used during ancient times, however, with the innovative technologies, it is recently possible to combine conventional fabric manufacturing techniques with a new generation of materials to create conductive fabric surfaces for wearable technologies. Moreover, yarn‐like electronic structures (traditional yarns, conductive yarns, conventional yarns which are functionalized with conductive materials, carbon nanotubes, etc.) can be woven or knitted in order to obtain E‐textiles products.^[^ ^70^, ^71^, ^72^, ^73^ ^]^ Knitted fabrics are more flexible than the woven ones, therefore where the stretchiness and flexibility are needed, knitting fabric structure would be an option, however, where the rigidity of the structure is required, woven fabrics would be a better option. Direct insertion of conductive yarns into the weaving and knitting manufacturing processes, sewing or embroidery systems are possible manufacturing routes for e‐textiles. Besides, coating, printing, and deposition of electroconductive solutions on the knitted, woven and nonwoven fabric surfaces are also alternative ways for the manufacturing of E‐textiles.^[^ ^74^, ^75^, ^76^ ^]^

For fabricating the woven fabrics with electrically conductive materials, conventional weave patterns are used. The combination of different types of conductive yarns and traditional polymeric yarns, both are used together in the weaving loom. The conductive yarns can be positioned either in weft or warp form in those woven structures in order to create electrical circuits.^[^ ^77^ ^]^ In this perspective, several studies have been carried out for the manufacturing of fabric‐based circuits by the weaving of conductive yarns. Such studies mainly focused on creating woven circuits for antennas, electromagnetic shielding, and USB applications.^[^ ^78^, ^79^, ^80^, ^81^, ^82^ ^]^ Conductive woven fabric with nickel addition and conductive knitted fabric which contains silver‐coated polyamide yarns are presented in Figure 3c,d.

Double layered woven fabric structures are the special types of woven fabrics. In a double layered woven structure, upper layer of warp yarns is connected with a bottom layer of weft yarns to create a durable and linked fabric structure. In this structure, conductive yarn can be embedded through those layers (as a middle layer) and hence by the insertion of conductive yarns in a hidden way, which prevents the possible short circuits through this layered structure.^[^ ^86^, ^87^ ^]^ Different types of conductive yarns (silver‐coated polyamide (PA)), stainless steel, and insulated copper yarns) were fed to the weaving process line together with conventional PET yarns in different weave patterns to create an electrical circuit in the structure with the aim of sensor integration. It is reported that; conductive yarn type and weaving type both are have considerable effects on the signal performance of the sensor integrated E‐textile structure.^[^ ^88^ ^]^

Indeed, multilayered woven and knitted structures offer a variety of different fabric pattern designs which may result in different sensing performances, for example, conductive yarn as an electrode is placed in the insulating layers of multilayered fabric structure in a sandwich forms.^[^ ^89^ ^]^ Using integrated textile connections in a form of multilayered structure where the conductive surface is shaped as a sandwiched between two textile layers, is an alternative to directly stitched conductive sensors on top of the fabric structures.^[^ ^90^ ^]^ Sandwich like structures containing conductive yarns in the middle assist the arrangement of the yarns into a skewed position thus causing gaps within the structure. When the pressure is applied to those structures, the gap between the layers is reduced and the conduction mechanism takes place hence pressure sensing circuit is completed.^[^ ^91^, ^92^ ^]^

Thanks to the nature of the knitted fabric, a single, conductive, continuous yarn provides structural continuity, which is followed through the all over the fabric and may be considered for the strain sensing applications. Depending on the knitted fabric elastic structure, the conductive yarn loops can come in contact and result in reduced resistance within textile material.^[^ ^93^ ^]^

Moreover, knittable conductive yarns were presented in the literature for their high capacitance performances. The reduced GO modified conductive yarns covered with a hierarchical structure of MnO_2_ nanosheets cooperated with polypyrrole thin film were used in the knitting machine to create capacitive textile fabrics for energy storage applications.^[^ ^94^ ^]^ Moreover, carbon fibers and carbon ink are brought together with knitting and screen printing technologies to create wearable textile‐supercapacitor electrodes.^[^ ^93^ ^]^

#### 2.3.2 Coating and Printing

The coating of textile material with a conductive polymer solution is one of the facile ways to turn the nonconductive textiles into the conductive structure. It gains great interest not only for the antistatic applications but also for other applications such as data transfer, sensing, monitoring and so on.^[^ ^95^ ^]^ Moreover, electrochemical coating of micro/nanofibers with conductive polymers is also well‐known technique.^[^ ^96^ ^]^ Apart from coating the fabric or yarn like structures with conductive polymer solutions, immersing the textile material into the conductive polymer solution is also a possible way of creating a conductive textile structure and this method is mainly used to change structure's capacitive nature. Moreover, conductive polymers solutions can also be used in the spinning process to obtain conductive fibers.^[^ ^20^, ^45^, ^97^, ^98^ ^]^ Furthermore, via in‐situ polymerization of conductive monomers (like pyrrole), it is possible to fabricate conductive polymer‐textile fabrics (cotton, linen, viscose rayon and polyester).^[^ ^99^, ^100^, ^101^ ^]^

Besides, the coating of conductive polymers can exhibit durability problems over time or else display inhomogeneity in the structure. For instance; electrically conductive PEDOT layers were covered on the textiles which are made of cotton and synthetic fibers via in‐situ polymerization. However, it was seen that their electrical properties are not stable toward the aging of the material and not homogeneous.^[^ ^102^ ^]^

Choi et al. introduced coating of poly(3,4ethylenedioxythiophene)/polystyrene sulfonate (PEDOT:PSS) film on the Kevlar yarn with the acidic treatment. It was reported that satisfactory results were obtained in terms of durability after exposure to air, repeated bending tests and washing.^[^ ^103^ ^]^ In another study, the different woven fabric containing nylon 6, poly(ethylene terephthalate) (PET), and poly(trimethylene terephthalate) (PTT) fabrics were coated with a conductive polymer (PEDOT) to achieve conductivity features. In this case, it was reported that PEDOT/nylon 6 composite fabrics have superior electrical conductivity (0.75 S cm^−1^) with poor resistance. Moreover, PTT/PEDOT composite structures presented better durability with moderate conductivity level (0.36 S cm^−1^).^[^ ^104^ ^]^ On the other hand, instead of direct coating of fabric, coating of a single fiber (nylon 6 or PET) with conductive polymer (PEDOT) or weave those coated fibers in the form of 3D woven fabric structure was also proposed to improve the quality.^[^ ^105^ ^]^

**Figure 4:**
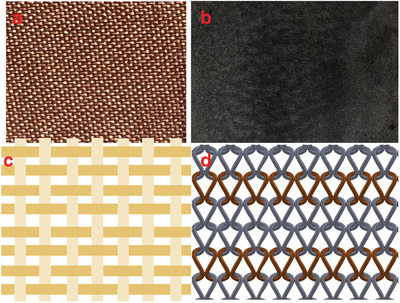
a) Photograph of Ni/Cu coated woven fabric. b) Photograph of PEDOT:PSS dip‐coated knitted cotton fabric. c) Basic weaving structure. d) Representation of knitted elastomeric and conductive yarn. Reproduced with permission.^[^ ^110^ ^]^ Copyright 2013, MDPI.

Conductive textile‐based materials can also be developed with screen printing or inkjet techniques using conductive inks. Different types of conductive inks are available in the market. The inks are composed of metallic solutions (Ag, Au, Co), conductive polymer solutions (polypyrrole, PANI, PEDOT) or nanoparticle (GO, CNT, graphene) containing solutions together with the addition of some surfactants, dispersants, thickeners, adhesion promoters, stabilizing agents.^[^ ^106^, ^107^, ^108^, ^109^ ^]^ **Figure** **4**a shows the cross‐section of Ag screen printed woven fabric and Figure 4b shows the PEDOT:PSS dip coated knitted fabric.

Conducting polymer solutions can be fed into printer as an ink where solidification of the conductive polymer solution has a key role to achieve the desired circuit parameters.^[^ ^111^ ^]^

Conventional textiles such as cotton and polyester fabrics were screen printed with porous carbon materials to obtain a flexible and lightweight fabric supercapacitor electrode.^[^ ^112^ ^]^ Knitted fabric structure including carbon fibers were screen printed with activated carbon ink to provide an energy storage ability.^[^ ^93^ ^]^ In another study, the application of the inkjet printing on the textile surface containing multiwalled carbon nanotube was investigated to produce flexible supercapacitor with certain flexibility.^[^ ^113^ ^]^

Without the usage of screens as in screen printing, ink‐jet printing is a promising printing method for creating electrically active textile materials in a one‐step agglomeration of the conductive layer on the substrate. Also, direct inkjet printing provides a cost efficient way to produce integrated circuits compared to photolithography or vacuum deposition.^[^ ^111^ ^]^ However, it has some challenges while applying it on textile material due to their porous and rough surface.^[^ ^114^ ^]^

Nano‐sized metallic particles were combined with the polymeric dispersions to obtain paste or ink for printing technologies. One of the most important parameters is to adjust the ink's viscosity with metallic solutions for direct printing applications. The main principle of metallic ink‐jet printing is that the metallic salt is fed to the system and in situ metal formation is occurred via redox reaction between reducing agent and metallic salt, thus metallic layers composed of aggregated metal particles are obtained on the printing surface such as paper, film or fabric.^[^ ^115^ ^]^ In the literature, metallic solutions were applied to the textiles via inkjet printing to create antennas. However, the main challenge for antennas from ink‐jet printing is to endure the system toward the bending forces.^[^ ^116^, ^117^, ^118^ ^]^ For capacitive sensor applications, ink‐jet printed silver electrodes were combined with PET fibers to create a dielectric layer.^[^ ^119^ ^]^

#### 2.3.3 Embroidery

**Figure 5:**
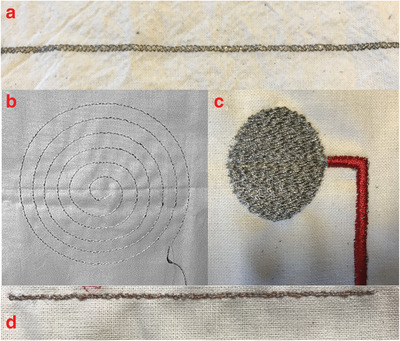
a) Embroidered silver yarn on the fabric. b) Embroidered stainless steel yarn as an antenna. Reproduced with permission.^[^ ^124^ ^]^ Copyright 2004, Elsevier. c) Embroidered textile electrode for ECG measurements. d) Embroidered copper yarns.

Embroidery is defined as a decorative arrangement of yarns, cords, beads, on the fabric or leather layer to obtain the desired configuration on the surface.^[^ ^120^ ^]^ Process flexibility of the embroidery is easily combined with the high‐performance fibers to create tailor‐made high performances materials.^[^ ^120^ ^]^ Moreover, digital embroidery designs allow combining electronic components (wires, switches, sensors or other electronics) on the textile surfaces. Conductive yarns can be embroidered with or without the conventional yarns to create an electrical conductivity feature on the textiles to let the current pass through embroidered design.^[^ ^121^, ^122^ ^]^ Stainless steel and polyester composite yarn were embroidered on the textile material to create electronic properties.^[^ ^123^ ^]^ Embroidered yarns can be used in the ECG measurements as an electrode or wearable antennas.^[^ ^124^, ^125^ ^]^ **Figure** **5** represents the different embroideries for the combination of electronic textiles. Metallic yarns and inherently conductive yarns can also be used in a tailored made circuit route on fabric via embroidery. In the literature, conductive polymeric yarns which are covered with PEDOT:PSS, were embroidered to have an E‐textile structure.^[^ ^126^ ^]^ Besides, creating a conductive pattern, embroidery can be used as an alternative to soldering and welding where they combine the textile substrate with the electronic components.^[^ ^127^, ^128^ ^]^

#### 2.3.4 Welding of Textiles

Welding can be defined as a joining or bonding technique that joins/bonds the metals and/or thermoplastics. This technique helps to combine the materials without using sewing thread. Whereas, sewing machines have limitations in terms of gathering different types of layers, sew‐free techniques such as bonding and welding have the edge on joining the wide range of materials.^[^ ^129^ ^]^ Generally, technical textiles, sportswear products, and underwear products benefit from the welding technologies. Sewing of textiles gathers the fabric layers by incorporating the thread and it is limited with thread length fed to the sewing machine so that it provides a certain amount of durability. However, the welding technique is an adequate alternative to join layers by directly applying pressure and/or heat on the contacting surfaces of fabric structure hence creating a continuous effect.^[^ ^130^ ^]^ Welding textile‐based products should withstand the corrosion, and easy to sew while preserving electrical conductivity properties of the conductive fabric structures.^[^ ^123^ ^]^ Welding and bonding textiles without the need for threads is an opportunity for getting a better performance for the joined layers.^[^ ^131^ ^]^ Welding can be done according to different phenomena's such as; thermal welding via conduction or convection capacity of materials, radiation (by the infrared, laser or induction), and friction welding by the rotary, vibrational, or ultrasonic. For textile‐based products generally, hot air fabric and hot wedge fabric welding, ultrasonic, laser, and radiofrequency welding techniques are used to weld or bond the materials.^[^ ^129^, ^131^, ^132^ ^]^ Welding is a good opportunity to combine layers or materials, particularly which are not suitable to sew with the sewing machine.

## 3 Characterization of E‐Textiles

Materials which are used for the formation of the E‐textiles structure should be examined by intrinsic high compliance, ease of their processability, ease of forming a shape and low weight to offer comfort for the wearer.^[^ ^133^ ^]^

Wearable E‐textile products should be stable enough toward the environmental conditions and have enough durability against the mechanical forces which are applied by the wearer and/or during their fabrication. In this perspective, physical and chemical examinations of E‐textile products are carried out likewise to the characterization methods of conventional textiles. However, the most important feature of E‐textiles, which is electrical properties, should be studied intensely for that new group of products.

### 3.1 Electrical Properties

In general, electronic textile‐based products exhibit lower dimensional stability compared to conventional electronic substrates. Electronic characterization of E‐textiles has some drawbacks due to their low dimensional stability, where they should ensure fabrication for safe and reliable electrical resistance. Surface resistivity characterization is one of the important electrical properties of textile‐based electronic products and it is relative to the contact of measuring device and textile sample, or reactivity of the environmental effects. Moreover, elasticity and deformability of textile‐based products affect the resistivity measurements and unstable conductive coatings also influence the electrical properties of the textile‐based materials.^[^ ^134^ ^]^

**Figure 6:**
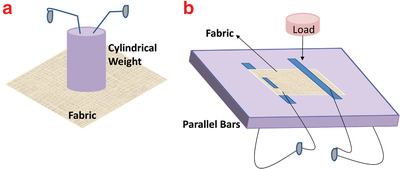
Schematic diagram of resistance measurement: a) cylindrical weight, b) parallel bars methods.

Possible ways to measure the electrical resistance of textile‐based materials are cylindrical weight, parallel bars, and sewn connections methods^[^ ^134^ ^]^ as shown in **Figure** **6**.

The resistivity (ρ) of conductive fabrics can be easily determined with resistivity meter by placing the fabric pieces in between the metallic disks under the controlled environmental conditions.^[^ ^98^, ^135^ ^]^ The resistivity (ρ) is defined as below Equation (1)

(1) $$\rho =2\pi \text{ln}\left(\frac{r1}{r2}\right)\;Rs$$

where ρ is surface resistivity, Rs denotes surface resistance, *r*1 and *r*2 indicate the radius of the outer and inner diameter of the ring electrode, respectively.^[^ ^135^ ^]^

Direct and alternating current (DC and AC, respectively) are electrical transmission sources while DC is commonly used for powering, AC is used for transmitting signal and sensing in the E‐textile circuits.^[^ ^127^ ^]^

The yarn is a complex structure that consists of fibers and/or filament entanglements to create a network as a fabric while the contacts of this network through the filaments are random. Mechanical deformations of textiles cause the change in charge path thus the deformation of conductive contacts vary due to the local deformation of textiles results in a variation in electrical resistance of strain fabric sensors.^[^ ^136^, ^137^ ^]^ For examination of the piezoresistive fabric sensors, mechanical characterization can be followed; it aims to examine the electrical response of the fabric strain sensor as a function of the external mechanical stimuli which is controlled by PC.^[^ ^136^ ^]^

The dielectric permittivity of the electrically conductive textile materials can be determined via the usage of known dimensions of two parallel capacitor plates with certain distance however, a variation on the results can be seen due to the 3D fabric structure which contains air as the ratio of it and yarns affects the dielectric permittivity of the fabric.^[^ ^138^ ^]^

For the E‐textile antennas, the low and the stable electrical resistance is required, surface resistance should be homogeneous and fabric should have the flexibility against bending and/or deformation. Thus, their electrical resistance and surface resistivity values have an important role.^[^ ^139^ ^]^ E‐textile antennas perform similarly as conventional antennas so that they can be examined with in the same route of conventional ones, moreover array deformation and bending have an important effect on the performance of E‐textile antennas.^[^ ^140^ ^]^

For investigating the long‐term durability and stability of the electrical contact behavior of conductive textiles, cycling load performance of the sample was combined with the electrical contact experiments. When conductive polymer coating on the ribbon has been damaged and cracks become more and more due to the applied force, the resistance of the conductive textile increased with further the cycling load.^[^ ^141^ ^]^ Similarly, E‐textile sensor stripes which are based on woven textiles were evaluated with the cyclic bend‐tester to determine the mechanical long‐term reliability of the E‐textile sensors while the stripes are attached in both sides and gap oscillates and conducts pneumatically.^[^ ^142^ ^]^

When textile substrates are compared with plastic boards, they have disadvantages in terms of their easily wrinkle nature and not dense as plastic boards to print on it. When the electronic printed circuit board is used on the textile substrate, the main parameter that affects the electrical resistance and the electrical stability of the film, wire or electrical junction is the roughness of the textile surface. To improve the resistance and to maintain the stability of the electrical connection, dense fabric with thin yarn is favorable to reduce the roughness of the surface.^[^ ^143^ ^]^ SEM image of the screen‐printed woven fabric is given in Figure 4a. Kim et al. studied the electrical performance of screen‐printed fabrics in terms of their DC resistance, AC impedance, and the maximal signal frequency with the time domain reflectometry profile.

Khumpuang et al. studied the contact resistance for PEDOT:PSS coated fibers with the contact of the fibers but the measurement apparatus applied a certain value of force and electrical contact is only recorded for the first measurement since the coating is damaged with the scratches after the first measurement so measurements could not be repeated.^[^ ^144^ ^]^

Investigation of their reproducibility and reliability electrical function of E‐textile products toward the conventional circuits were also carried out. In addition to E‐textiles' electrical properties, their mechanical properties together with its textile physical properties should be also deeply investigated to create feasible products.^[^ ^145^ ^]^

### 3.2 Textile Physical and Chemical Properties

Textile characterization methods can be investigated from two aspects the first deals with the property‐based test methods which give us information about the material's natural physical and chemical structure and the second is the performance‐based test methods which provide information about service stage, and responses of the material during its usage.^[^ ^146^ ^]^ For E‐textile, both methods are investigated to determine the properties of the E‐textile samples. Textile characterization methods should be done in‐depth to understand the structure because generally, textiles are complex structures. For that reason, several well‐known organizations such as ISO, ASTM, CEN, and AATMC have published standards to follow during the examination of textiles.^[^ ^147^ ^]^ In the case of necessity, all conventional mechanical test methods such as stress‐strain tests can be followed for the conductive material included in textile‐based samples. For wearable E‐textile products, the main expected properties are flexibility and resistance against forces occurring during their fabrication. Besides, during their usage, shear and bending rigidity, resistance to chemicals, breathability, and resistance to washing are also gaining importance.

With regard to the development of technological tools, optical microscopy analysis gave way to enhanced microscopic examination apparatus. The light microscope helps to investigate the external structure of bulk and fibrous polymers, whereas the transmission electron microscope (TEM) provides information about ultrastructural parts of the sample.^[^ ^148^ ^]^ For the examination of morphological properties of E‐textile component scanning electron microscopy (SEM) and atomic force microscopy (AFM) are well‐known techniques to record surface properties and have better resolutions when they are compared to conventional optical light microscopy. AFM is used for areas that are limited in size but SEM can analyze larger surfaces which means that with SEM it is possible to observe the fabric surface whereas AFM can be used to determine the fiber surface. Using these techniques, it is possible to demonstrate conductive polymer deposition on the surface level and examine the homogeneity of the covering.

Wearable E‐textile products should be within the scope of the wearable design for the convenience of the user; they should be flexible enough while still being robust through external and internal forces. Thus, the conventional stress‐strain experiment can be followed to investigate the product's mechanical properties. Generally, textile products consist of a porous structure therefore, the porosity of the E‐textile has an important role in enduring the comfort feature.

Moreover, within the scope of wearability, main performance requirements such as, breathable, lightweight easy to wear, comfort, launderability strength, abrasion and so on have been investigated for wearable electronic textiles.^[^ ^87^ ^]^ As described in the previous sections, the stability of the conductivity feature of textile‐based materials is affected by light, abrasion, washing, chemical treatments and washing with detergents.^[^ ^149^, ^150^ ^]^ Throughout the product's usage, the reduction of their level of conductivity is inevitable.^[^ ^30^ ^]^ Research is still going on to ensure solutions for long‐life E‐textile products which will be reliable enough for the wearers in terms of functioning, and are resistant to mechanical and chemical factors while providing comfort and flexibility.

## 4 Integration Strategies for E‐Textiles

Creating conductive flexible materials is not enough individually to build wearable E‐textiles. They should also be well integrated into the garments for functionality. The attachment of sensing elements within textiles using embedded processes offers long term monitoring without deterioration in comfort.^[^ ^151^ ^]^ There should be interconnections between conductive yarns/fibers and electronic components such as sensors and switches in order to transfer signals and therefore requiring robust connections.^[^ ^152^ ^]^ The main scope of the interconnection of E‐textile substrates is to transmit signals without losing their quality, thus several methods are suggested in the literature to integrate E‐textile components. This can be accomplished by way of physical and/or mechanical bonding or the usage of conductive adhesives.

**Figure 7:**
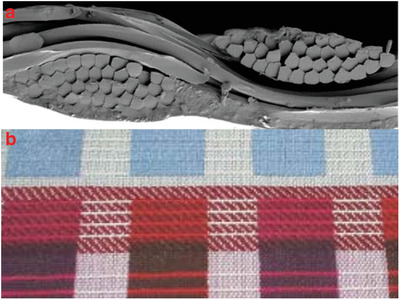
a) Cross‐section of woven fabric. Reproduced with permission.^[^ ^28^ ^]^ Copyright 2018, MDPI. b) Photograph of the woven optoelectronic fabric. Reproduced with permission.^[^ ^153^ ^]^ Copyright 2013, Elsevier.

Conductive threads which are made out of conductive metals, metallic‐coated or metal‐wrapped yarns, or conductive polymers are used to weave circuits, sensor or switches on the fabric directly (**Figure** **7**b).^[^ ^153^ ^]^ Also, Figure 7a shows the cross‐section of woven fabric. Moreover, conductive yarns are directly woven into the fabric for ultrasonic sensor integration.^[^ ^86^ ^]^

Metal filaments and yarns made from steel, aluminum, copper, brass or nickel are mainly used to create electronic fabrics. Fabrics that are combined with metals are low cost and have good strength with reliable electro‐conductivity. However, they are not as flexible as polymer‐based textiles. The deformation of metals cannot be easily recovered and those structures are heavy and bulky often rendering the fabric uncomfortable.^[^ ^54^, ^78^, ^95^, ^154^, ^155^ ^]^

Electrically conductive adhesives (ECAs) are also used to connect the electronic components with textile base materials. ECAs are created to replace the tin‐lead solder in electronic circuits.^[^ ^152^ ^]^ ECAs consist of two parts: one is polymeric (epoxy or silicone) providing enough mechanical properties to the system, the other is metal/conductive fillers (silver, gold, nickel, copper or carbon, etc.) providing conductivity.^[^ ^156^ ^]^ When choosing an integration system for E‐textile products, ECAs can be a good choice to attach yarns/fibers to the other electronic components due to their nontoxicity, durability, and tailor‐made properties according to its area of application or end‐use.^[^ ^157^ ^]^ Another possible route for integration is a combination of adhesive bonding and embroidery to connect the electronic parts of the system to the fabric.^[^ ^158^ ^]^

Fabrics which consist of thermoplastic materials in terms of yarns or coating are more suitable for welding.^[^ ^159^ ^]^ Textile materials can be welded using hot air, hot wedge, ultrasonic, laser and/or radiofrequency welding techniques for connectivity.^[^ ^160^, ^161^, ^162^ ^]^

From the E‐textile point of view, steel yarn is welded on the substrate and encapsulated with epoxy to enhance the conjunction, however, it is affected by the adhesion of the thread through pull strength.^[^ ^123^ ^]^ For instance, research has shown that an appropriate combination of welding parameters with the connection of textile transmission lines, while still preserving the textile aspects is possible using the hot air welding method.^[^ ^131^, ^160^ ^]^ Moreover, in medical applications, where the connections should be biocompatible, welding can be a good alternative over soldering.^[^ ^162^ ^]^

## 5 Application Areas of Wearable E‐Textiles

**Figure 8:**
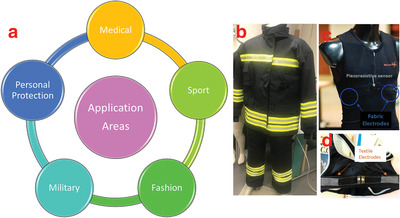
a) Main application areas of wearable electronic textiles. b) Firefighter suit equipped with emergency response sensors. c) Wearable piezoresistive sensors and fabric electrodes. Reproduced with permission.^[^ ^193^ ^]^ Copyright 2016, MDPI. d) Image of the wearable knitted electrodes for the measurement of ECG signal. Reproduced with permission.^[^ ^167^ ^]^ Copyright 2018, MDPI.

Wearable electronic textiles are an increasingly expanding field which is raising value for both academicians and industry. They present wide range of applications for such as energy storage materials,^[^ ^163^ ^]^ monitoring, textile‐based antennas, energy harvesting and biosensor applications, sweat sensors, health care systems, sports and entertainment products.^[^ ^164^, ^165^, ^166^ ^]^ **Figure** **8** shows the selected example products of wearable electronic textiles.

One of the most common and fastest increasing application is medical fabric electrodes, due to their ease of use and simple integration with daily use products such as underwear or belts, which does not require an external gel or cable connection. Fabric‐based electrodes are used to monitor the electrocardiogram (ECG) signal as an alternative to conventional Ag/AgCl electrodes^[^ ^136^, ^167^ ^]^ PEDOT:PSS electrodes are a suitable alternative to conventional Ag/AgCl electrodes. Several publications show that combined electronic and ionic conductivity properties of PEDOT and PSS present improvements in the textile‐based ECG electrodes.^[^ ^168^, ^169^, ^170^ ^]^ Moreover, PEDOT:PSS based textile electrodes can be used to determine the surface electromyography (sEMG).^[^ ^171^ ^]^

Together with the modern life rituals flexible batteries and supercapacitors gained more popularity due to their adequate power supply, lightweight, portable and wearable features. It is common to see E‐textiles in biomedicine applications particularly healthcare products build a bridge between humans and wearable devices.^[^ ^172^, ^173^ ^]^ Thanks to the developing technologies, it is possible to monitor several vital signals from humans via E‐textile products, for instance, heart rate, electrocardiogram, respiration rate, skin temperature.^[^ ^8^, ^174^ ^]^

Flexible energy storage devices can be 1D (linear) or 2D (planar) structures; polymer films, metal sheets, and textile‐based products are examples of planar supercapacitors.^[^ ^56^, ^175^, ^176^ ^]^ Metal substrates, which are used in capacitive applications give excellent conductivity, however, they are poor from the flexibility and not lightweight. While adapting materials to the system, the ultimate volumetric performance should also be taken into an account.^[^ ^57^ ^]^ Carbon fabrics that are made from CFs are promising candidates for supercapacitor applications due to their flexibility, chemical resistance and high surface area because of their 3D structures. Moreover, activated carbon fiber can be directly used as an electrode for supercapacitor application.^[^ ^57^ ^]^ There are also further studies about increasing the activity of (activated carbon fiber) ACF electrodes, by coating the ACFs with carbon materials, metallic oxides or conducting polymers for increasing the electrical conductivity. Carbon nanofiber webs are the other carbon‐based textile products that are used in supercapacitor applications due to their higher surface area and lightweight and chemical resistance.^[^ ^177^, ^178^, ^179^ ^]^ An alternative to conventional conductive yarn applications nanofiber patch in the cloth with conductive yarns can be used as a heart rate and electrocardiogram sensor due to its improved adhesion to the skin, which improves the stability of the measurement of the biomedical signals.^[^ ^180^ ^]^ Structural health monitoring (SHM) is a tracking system of structure through time. It is based on a state of structure that should remain stable through time. Thanks to the monitoring system of SHM at time axis; damages, lifetime assumptions, etc. can be detected^[^ ^181^ ^]^ using textile‐based sensors. Carbon filament yarns exhibit lightweight and can be used as a reinforcement inside the thermoplastic composite structures, which makes them an appropriate candidate in the structural health monitoring of textile composites as a strain sensor.^[^ ^182^, ^183^ ^]^ As a textile material, fabric‐based composite structures could be used as a skeleton for the placement of miniaturized sensor for structural health monitoring.^[^ ^184^, ^185^ ^]^

Energy storage devices, mainly lithium‐ion batteries and supercapacitors provide us an operational structure from electrochemical energy sources.^[^ ^186^ ^]^ These systems can be good alternatives for supplying power to wearable electronics.^[^ ^187^ ^]^ Paper‐based batteries also have several advantages and are gaining more popularity due to their lightweight, high surface area and easy integration with power system improves autonomy time and provide high power capacities.^[^ ^188^ ^]^ In comparison to conventional batteries, textile‐based batteries are nontoxic and can be combined with thermoelectric^[^ ^189^ ^]^ or solar panels^[^ ^163^, ^190^ ^]^ to assist in harvesting the energy from sunlight and/or body movements and heat, however, the generated power is still limited and not enough for applications.

Development in nano and micro‐technologies can be attributed to reduction in power consumption of systems, or self‐powered systems, which is resulting in increased portable electronics and biomedical applications.^[^ ^191^ ^]^

The ordinary human body is a source of power on its own and provides an opportunity to harvest energy even from the basic movements of human such as breathing. For this reason, energy applications based on human motions can be promising for wearable electronic applications.

In the personalized healthcare products, wearable electronics have a principal role to monitor the physiological and biomechanical signals of the human body with flexible and lightweight materials instead of bulky devices.^[^ ^182^ ^]^ Zhong et al. have recently developed a fiber‐based generator that can convert biomechanical motions/vibration energy into electricity by the electrostatic induction effect using a modified cotton thread coated with only CNT and polytetrafluoroethylene (PTFE). Those yarns were woven together to create power shirts for wireless body temperature system.^[^ ^192^ ^]^

Till now, a range of prototypes were introduced in the electronic textiles area such as self‐illuminating handbag interiors, illuminating clothes or gym kits which reflect the workout performance.^[^ ^153^ ^]^ Italian company Luminex manufactured a fabric full of LEDs (light‐emitting diodes) attached to it which turns the clothing into a glittery dress. Those LED fibers were powered by small rechargeable batteries and the tailor‐made dress was designed with a switching button option that let the user glow her dress in one of five different color options.^[^ ^153^ ^]^

Organic light‐emitting diodes (OLEDs) from fibers for wearable E‐textiles were introduced with properties of flexibility, comfort, and being durable/ and washable. The organic light‐emitting diodes (OLEDs) were manufactured based on a dip coating technique of PET fibers.^[^ ^194^ ^]^

Fabric electrodes, which are fabricated to create a health monitoring system were produced via knitting technology. Conductive stainless‐steel yarns (which were twisted around the viscous yarn) were knitted via tubular intarsia technique to form electrodes in a fabric structure and double face fabric was formed for the desired local internal conductive areas.^[^ ^151^ ^]^

For the potential WLAN applications of E‐textiles, Ouyang et al. suggested that for E‐textile antennas, satin weave fabrics represent better performance than plain weave fabrics due to the variances on its front and back sides. In their study, they suggested E‐textile antenna structure presenting 6.59 dB and bandwidth of 3.4% at 2.44 GHz which is suitable for the WLAN communications and they also reported that they can be alternative to fully metallic textile patch antennas.^[^ ^195^ ^]^

There are several applications of electronic textiles due to their pressure sensing properties.^[^ ^196^, ^197^, ^198^ ^]^ The main principle of creating pressure sensors based on an E‐textile structure is to use conductive fabrics as an electrode together with resistive material in‐between layers. Therefore, the connections among contacts between conductive parts create resistance change when the pressure is applied. The sensor has a similar touch and feels like the conventional fabrics so that they can be easily embedded into clothing or beddings.^[^ ^199^ ^]^ In a study, the fabric touch sensor was created by a woven fabric structure in which the conductive polymeric yarns were used. Fabric touch sensors have enough pressure sensibility to be used in human touched systems such as wearable keyboards or health care systems.^[^ ^200^ ^]^ The E‐textile based pressure sensors have a wide range of examples in the sports field such as skiing, taekwondo. During skiing, it offers to detect the posture of the skier whereas in taekwondo it is possible to detect the impacts of punches or kicks via protection vest including a pneumatic system as a form of the wearable body protector.^[^ ^201^ ^]^ In another study, a pressure sensor was created in the form of a sandwich structure composed of Nylon fabric plated with Tin/Copper/Silver coated yarns for sports rehabilitation or ergonomic products.^[^ ^202^ ^]^

## 6 Future Studies and Conclusions

Wearable electronic products present exciting possibilities together with personalized algorithms. Throughout the review, their fabrication techniques and usage areas are investigated. Electronic wearable textiles attract a great deal of interest due to their easy and flexible usage in our daily lives under a wide variety of applications. Possible product groups of E‐textiles can be divided into five main groups: Sport, medical, fashion, personal protective equipment, and military areas. Common characteristics of all these five groups should fulfill the basics of textile material properties in the form of E‐textile products, such as laundering, stretching and flexing. Especially, we can add other characteristics according to the demanded area, like abrasion, UV exposure so on. Electronic textiles should offer durability, viability and economic power consumption for the long life cycle. Moreover, they should be reliable for functioning. Washing performance of E‐textiles is critical to commercial success. Polymer coating or application of encapsulation on E‐textile structures' is also critical to provide enough strength and durability to the washing and environmental conditions for daily usages. However, studies should be furthered for compact solutions. Hopefully, the washing performance of smart textile materials is promising for the coming future. Industrial scale production of the new generation technologies such as wearable electronic textiles needs to be discussed throughout the academic‐industry collaborations. Washing performance will be critical to the commercial success of intelligent textiles since only a single‐usage is an expensive way. Industrial production of the new generation technologies for wearable electronics needs to be further discussed as a new potential research area, since the mass production of E‐textiles is still a challenging issue and has not been discovered yet.

## Conflict of Interest

The authors declare no conflict of interest.
